# Switch telomerase to ALT mechanism by inducing telomeric DNA damages and dysfunction of ATRX and DAXX

**DOI:** 10.1038/srep32280

**Published:** 2016-08-31

**Authors:** Yang Hu, Guang Shi, Laichen Zhang, Feng Li, Yuanling Jiang, Shuai Jiang, Wenbin Ma, Yong Zhao, Zhou Songyang, Junjiu Huang

**Affiliations:** 1 Key Laboratory of Gene Engineering of the Ministry of Education and Institute of Healthy Aging Research, School of Life Sciences, Sun Yat-sen University, Guangzhou, 510275, China; 2 Key Laboratory of Reproductive Medicine of Guangdong Province, School of Life Sciences and the First Affiliated Hospital, Sun Yat-sen University, Guangzhou, 510275, China

## Abstract

Activation of telomerase or alternative lengthening of telomeres (ALT) is necessary for tumours to escape from dysfunctional telomere-mediated senescence. Anti-telomerase drugs might be effective in suppressing tumour growth in approximately 85–90% of telomerase-positive cancer cells. However, there are still chances for these cells to bypass drug treatment after switching to the ALT mechanism to maintain their telomere integrity. But the mechanism underlying this switch is unknown. In this study, we used telomerase-positive cancer cells (HTC75) to discover the mechanism of the telomerase-ALT switch by inducing telomere-specific DNA damage, alpha-thalassemia X-linked syndrome protein (ATRX) knockdown and deletion of death associated protein (DAXX). Surprisingly, two important ALT hallmarks in the ALT-like HTC75 cells were observed after treatments: ALT-associated promyelocytic leukaemia bodies (APBs) and extrachromosomal circular DNA of telomeric repeats. Moreover, knocking out hTERT by utilizing the CRISPR/Cas9 technique led to telomere elongation in a telomerase-independent manner in ALT-like HTC75 cells. In summary, this is the first report to show that inducing telomeric DNA damage, disrupting the ATRX/DAXX complex and inhibiting telomerase activity in telomerase-positive cancer cells lead to the ALT switch.

Continuous telomere loss which derives from DNA replication, drives the fusion of chromosome ends^1^, leads to cell cycle arrest and induces cell senescence^2^,^3^. However, tumour cells can maintain telomere length and proliferation through telomerase reactivation or the alternative lengthening of telomeres (ALT) mechanism^4^,^5^. It is reported that approximately 85–90% of cancer types are telomerase-positive, which use its RNA subunit (termed TR or TERC) as a template and its telomerase reverse transcriptase (TERT) to maintain chromosomal end by adding 5′-GGTTAG-3′ hexanucleotides^6^,^7^. Due to lack of telomerase activity in human somatic cells, telomerase is considered as a potential target of cancer therapy. However, this strategy would be ineffective in several human cancers^8^,^9^,^10^, which are lack of detectable telomerase activity and utilize the ALT mechanism relying on recombination-mediated telomere elongation^5^,^11^,^12^,^13^. Previous studies have shown that anti-telomerase therapy provoked a switch from telomerase activity to the ALT mechanism in mice^14^,^15^,^16^. Furthermore, it has been shown that the ALT is an alternative mechanism for telomere maintenance during oncogenesis^10^, which would ultimately decrease the effectiveness of anti-telomerase treatment. Therefore, identifying the mechanism of ALT induction and the telomerase-ALT switch is beneficial in resolving the bottlenecks of anti-telomerase therapy^9^,^17^.

ALT-positive cells typically contain abnormally heterogeneous telomeres, ALT-associated promyelocytic leukaemia bodies (APBs) and extrachromosomal TTAGGG repeats (ECTRs)^18^,^19^. Despite understanding the hallmarks of ALT, the mechanism of ALT induction remains unknown. The study of ALT activation which transformed a telomerase-positive cell line into an ALT-positive cell line *in vitro* is rare^20^,^21^. Recently, several factors have been shown to contribute to ALT formation. It has been reported that the depletion of a histone chaperon ASF1 resulted in ALT cells induction and long telomeres elongation concomitant with inhibition of telomerase activity^20^. Some factors can suppress ALT mechanism. When ALT cells fused with telomerase-positive cells and somatic cells, the ALT phenotype was suppressed^22^,^23^. The loss of ALT activity in these hybrid cells suggested that ALT repressors might exist in telomerase-positive cells and somatic cells^22^. Although telomerase and ALT activity can coexist in human cells, some ALT phenotypes, such as heterogeneity of telomere length, are inhibited^15^,^22^,^23^,^24^,^25^. Firstly, since the ALT mechanism is a recombination-mediated lengthening mechanism, the clustering of telomeres caused by DNA damage response (DDR) promotes homology-directed telomere synthesis, suggesting that DDR may play an important role in ALT induction^26^,^27^,^28^,^29^. Secondly, somatic mutations of the histone variant H3.3, alpha-thalassemia X-linked syndrome protein (ATRX) and death associated protein (DAXX) have been found in ALT cancers, including pancreatic neuroendocrine (panNET) cancers and glioblastoma multiforme (GBM) cancers^30^,^31^. They are chromatin remodeling factors at telomeres, which are responsible for ALT activity^32^,^33^. Furthermore, it has been shown that ATRX inhibits ALT and relates to telomerase assembly and depositing^21^,^34^,^35^. Although single and double deletion of ATRX and DAXX could not initiate the ALT mechanism, histone management dysfunction and chromatin structure disorder might provide a suitable genomic environment for ALT induction^20^,^31^,^32^,^36^,^37^. Thirdly, telomerase activity plays very important role in ALT repression. Inhibition of telomerase activity might promote ALT induction^14^. It has been shown that genetic extinction of telomerase in T cells of ATM knockout mice results in tumor emergence, concomitant with the increase of APB and C-circles^14^.

To determine the mechanism by which telomerase-positive cancer cells switch to ALT and to elucidate the mechanism of ALT induction, we induced telomere-specific DNA damage, disrupted the function of the ATRX/DAXX complex and inhibited telomerase activity in telomerase positive cancer cells, which successfully transformed a telomerase-positive cell line into a ALT-positive cell line.

## Results

### The establishment of TPP1^ΔOBRD^, ATRX knockdown or/and DAXX deletion telomerase-positive cell lines

**Figure 1:**
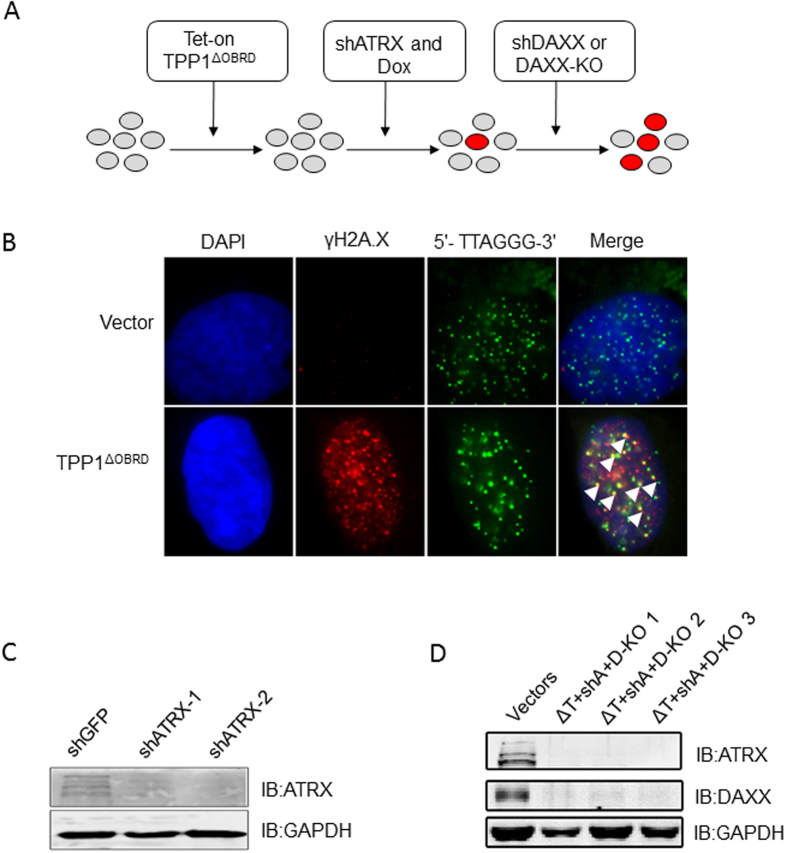
The establishment of the different cell lines. (**A**) Strategy for construction of the different cell lines. The red circles indicated the potentially transformed cells. KO, knock out. (**B**) IF-FISH was carried out to detect co-localization of γH2A.X (red) and telomeric (5′-TTAGGG-3′)_3_ probes (green) in the TPP1^ΔOBRD^ overexpressing cells with 100 ng/ml of doxycyclin. We used DAPI staining (blue) to indicate the cell nuclei. The white arrowheads showed the co-localization of γH2A.X and telomeres. (**C**) The knockdown efficiency of ATRX shRNAs were measured using anti-ATRX antibody for western blotting. GAPDH was used as the loading control. shATRX-1, ATRX shRNA1; shATRX-2, ATRX shRNA2. (**D**) The knockdown efficiency of ATRX and DAXX in three ΔT + shA + D-KO cell lines was detected using anti-ATRX antibody and anti-DAXX antibody for western blotting. GAPDH was used as the loading control. ΔT, TPP1^ΔOBRD^; shA, ATRX shRNA2; D-KO 1, D-KO 2 and D-KO 3 indicated the three DAXX knock out clones.

Cell line construction approach is shown in Fig.1A. Deletion of the OB-fold domain and the RD domain of TPP1 (TPP1^ΔOBRD^) partially suppressed telomerase activity and induced telomeric-specific DNA damage^38^,^39^. First, we constructed TPP1^ΔOBRD^ expressing telomerase-positive human fibrosarcoma cells (HTC75). Because long-term expression of TPP1^ΔOBRD^ might be lethal, we used a Tet-on inducible expression system to control TPP1^ΔOBRD^ expression^40^. After treatment with doxycycline (DOX), the HTC75 cells with high levels of TPP1^ΔOBRD^ expression showed significantly increased telomeric-specific DNA damages by detecting the signal of γH2A.X (Fig. 1B). Moreover, western blotting (WB) analysis showed that TPP1^ΔOBRD^ was expressed in the treatment of DOX (Fig. S1A). Next, we selected efficient shRNAs to induce ATRX or DAXX knockdown (Fig. 1C and Fig. S1B,C). WB results showed that both shATRX-1 and shATRX-2 could knockdown ATRX sufficiently, specifically shATRX-2 (Fig. 1C and Fig. S1C). Therefore, we planned to use cell lines expressing TPP1^ΔOBRD^, shATRX-2 (shA) and DAXX shRNAs for the subsequent experiments. However, we observed that the knockdown efficiency of both shDAXX-1 and shDAXX-2 were decreasing in late cell passage. To solve this problem, we used the CRISPR/Cas9 technique to generate TPP1^ΔOBRD^ (ΔT), shA and DAXX knock out (D-KO) HTC75 cell lines (termed by ΔT + shA + D-KO, Fig. S2). The expression level of ATRX and DAXX proteins in three ΔT + shA + D-KO HTC75 cell clones were nearly undetectable by WB (Fig. 1D). In conclusion, we successfully established different TPP1^ΔOBRD^, ATRX or/and DAXX deletion telomerase-positive HTC75 cell lines for further studies.

### TPP1^ΔOBRD^, knockdown of ATRX and DAXX significantly induced APBs formation in telomerase-positive cancer cells

**Figure 2:**
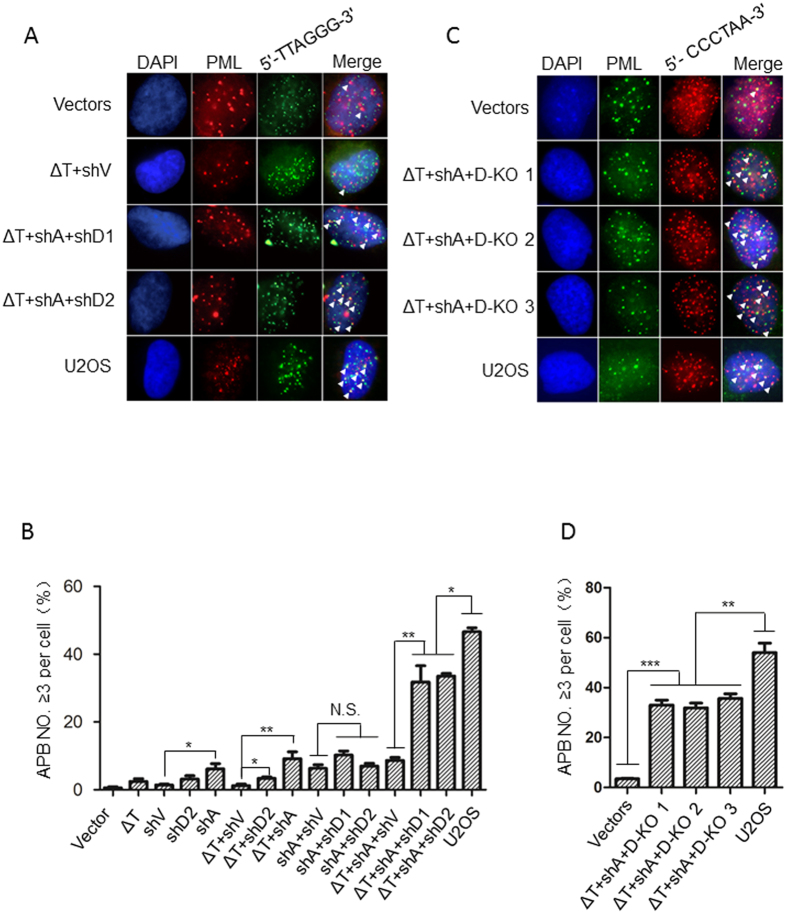
APBs detection in the different cell lines. (**A**) Immunofluorescence and fluorescent *in situ* hybridization (IF-FISH) staining for different cell lines. The PML bodies are labelled red. The telomeric DNA was labelled with (5′-TTAGGG-3′)_3_ probes (green). The nuclei were stained with DAPI (blue). The white arrowheads indicated PML and telomeric DNA co-localization. (**B**) Statistical quantification of (**A**), it indicated that the percentage of cells with more than three APBs. The error bars represented the standard error of three distinct experiments. *P < 0.05; **P < 0.01; ***P < 0.001; N.S., not significant. (**C**) IF-FISH staining. The PML bodies were labelled green. The telomeric DNA was labelled with (5′-CCCTAA-3′)_3_ probes (red). The nuclei were stained with DAPI (blue). The white arrowheads indicated PML and telomeric DNA co-localization. (**D**) Statistical quantification of (**C**), it indicated that the percentage of cells with more than three APBs. The error bars represent the standard error of three distinct experiments. **P < 0.01; ***P < 0.001. ΔT, TPP1^ΔOBRD^; shV, shRNA empty vector; shA, ATRX shRNA2; shD1, DAXX shRNA1; shD2, DAXX shRNA2; D-KO 1, D-KO 2 and D-KO 3 indicated the three DAXX knock out clones.

In ALT cancer cells, ALT-associated promyelocytic leukemia (PML) bodies specifically localize to telomeres to form ALT-associated PML bodies (APBs), which are important for recombination-based telomere elongation^18^,^41^,^42^. To determine whether TPP1^ΔOBRD^ overexpression, knockdown of ATRX or/and DAXX could transform telomerase-positive HTC75 cells into ALT-like cells, we used immunofluorescence and fluorescent *in situ* hybridization (IF-FISH) to detect APBs formation in different cell lines. In control HTC75 cells, we observed abundant PML signals, yet few were associated with telomeric DNA (Fig. 2A). The overexpression of TPP1^ΔOBRD^ (ΔT) or shDAXX-2 (shD2) did not affect the formation of APBs, but shATRX-2 (shA) itself significantly increased APBs (Fig. 2B). Also shA + shD1 (shDAXX-1) and shA + shD2 promoted the formation of APBs, which was similar to ΔT + shA, comparing to ΔT + shV (shVector) (Fig. 2B). Interestingly, in ΔT + shA + shD1 and ΔT + shA + shD2 cells, APBs were increased significantly, which were higher than the ΔT + shV cells and the ΔT + shA + shV cells, but still lower than human ALT osteosarcoma U2OS cells (Fig. 2A,B). Because DAXX shRNAs were unable to completely knock down the endogenous DAXX protein (Fig. S1B), we used the CRISPR/Cas9 technique to produce three ΔT + shA + D-KO cell lines (Fig. 1D). Using IF-FISH to detect the formation of APBs, we found that all three cell lines (ΔT + shA + D-KO1/2/3) had much more APBs formation, which were similar to the level observed in the ΔT + shA + shD1cells and ΔT + shA + shD2 cells (Fig. 2A–D).

### TPP1^ΔOBRD^, knockdown of ATRX and DAXX significantly induced the formation of C-circles and elongated telomeres in the telomerase-positive cancer cells

**Figure 3:**
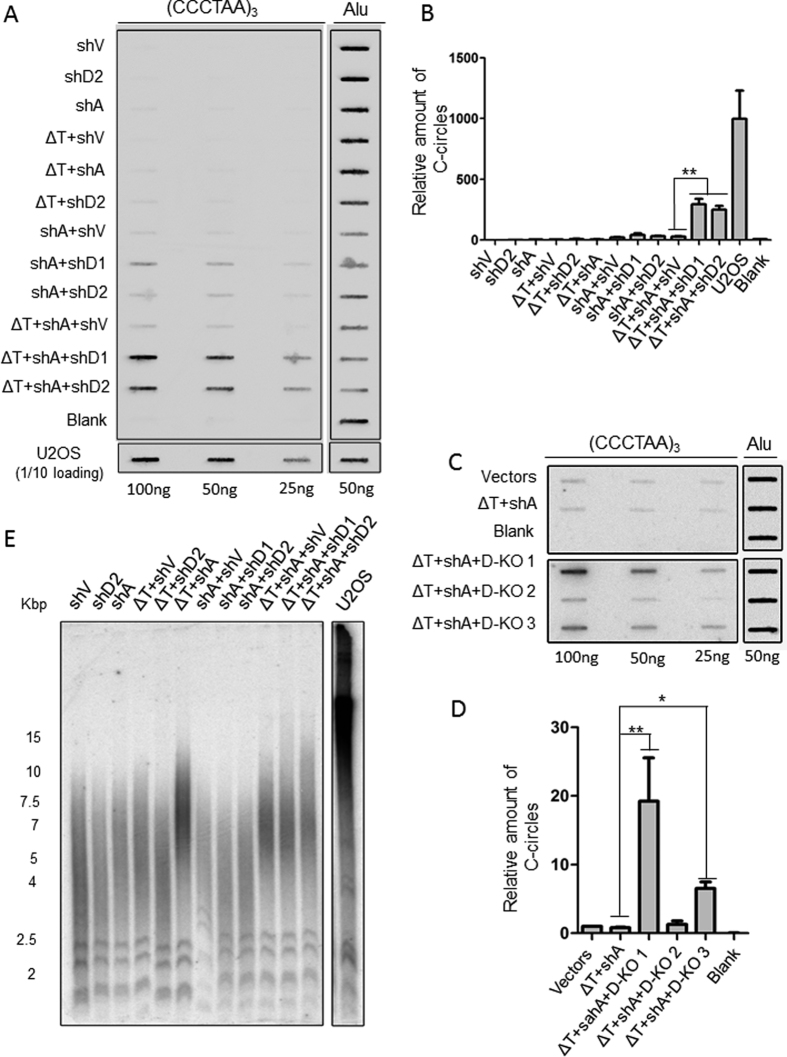
The detection of C-circles and telomere length in the different cell lines. (**A**) Different amounts of genomic DNA (100 ng, 50 ng, 25 ng) from the control cells and the different induced cell lines were used for the C-circles assay using biotin labeled telomeric (5′-CCCTAA-3′)_3_ probes and detected by chemiluminiscence. Due to the C-circles of U2OS is higher level, one-tenth of U2OS sample was loaded. A total of 50 ng of genomic DNA was detected using ALU probes as the input control. U2OS cells were used as the positive control and HTC75 cells with vectors were used as the negative control. The reactions which phi29 was omitted were the control for C-circles assay. (**B**) Statistical quantification of (**A**). The intensity values of the shV control bands were normalized to 1. The ratio of each group vs. the control group was calculated for all three DNA concentrations. The values were then combined and averaged for each cell line to determine the relative C-circle formation activity. Error bars indicated standard errors (n = 3). **P < 0.01. (**C**) Different amounts of genomic DNA (100 ng, 50 ng, 25 ng) from the control cells and the different induced cell lines were used for the CC assay using ^32^P-labeled (5′-CCCTAA-3′)_3_ probes. A total of 50 ng of genomic DNA was detected using Alu probe as the input control. The reactions which phi29 was omitted were the control for C-circles assay. (**D**) Statistical quantification of (**C**). The data analysis was performed as described in (**B**). *P < 0.05,**P < 0.01. (**E**) Telomeric Terminal Restriction Fragment (TRF) assay was used to analyse the telomeric DNA length in the different cell lines. ΔT, TPP1^ΔOBRD^; shV, shRNA empty vector; shA, ATRX shRNA2; shD1, DAXX shRNA1; shD2, DAXX shRNA2; D-KO 1, D-KO 2 and D-KO 3 indicated the three DAXX knock out clones.

APBs are important for telomere elongation through recombination in ALT cancer cells. In addition to telomere recombination, circular extrachromosomal TTAGGG repeats (ECTRs) production also induces telomeric DNA instability. One type of ECTRs, C-circles, are partially single-stranded telomeric (CCCTAA)_n_ DNA that are specific and quantifiable markers of ALT activity, which can be detected using C-circles assay (CC assay)^43^. To determine whether overexpression of TPP1^ΔOBRD^, knockdown of ATRX and DAXX in HTC75 cells leads to the formation of C-circles, we used a CC assay to analyse the level of C-circles in these different cell lines. Consistent with the APBs results, we detected significantly increase of C-circle signals in the ΔT + shA + shD1 cells and ΔT + shA + shD2 cells, which were not observed in the other cell lines (Fig. 3A,B). The C-circles were also increasing in ΔT + shA + D-KO 1 and ΔT + shA + D-KO 3 cells (Fig. 3C,D). However, ΔT + shA + D-KO 2 cells only showed a slightly increase of C-circles than the ΔT + shA control.

Due to the presence of both APBs and C-circles, which are two main characteristics of ALT cells, we concluded that the TPP1^ΔOBRD^, ATRX knockdown and DAXX deletion cells were ALT-like cells. ALT cells typically contain heterogeneous telomeres compared with telomerase-positive cancer cells. Therefore, we assessed the telomere length of the ALT-like cell lines at twenty population-doubling (PD20) using telomeric terminal restriction fragment (TRF) analysis. We observed telomeres elongation in the ΔT + shV cells, ΔT + shA cells, ΔT + shA + shV cells, ΔT + shA + shD1 cells and ΔT + shA + shD2 cells, but their pattern of telomere length distribution were still different from ALT U2OS cells (Fig. 3E). It was not a surprise that telomeres were elongated in these cell lines, since overexpression of TPP1^ΔOBRD^ could lead to telomere length elongation^44^. We found that telomerase were still activated in these cell lines (Fig. S3A,B). As telomerase suppresses the heterogeneity of telomere length in the ALT-like cells^15^,^22^,^23^,^24^,^25^,^45^,^46^, this might be the main reason why we could not find heterogeneous telomeres in these constructed cell lines.

### Deletion of hTERT in the TPP1^ΔOBRD^, ATRX knockdown and DAXX KO cells significantly increased heterogeneity of telomeres in ALT-like cells

**Figure 4:**
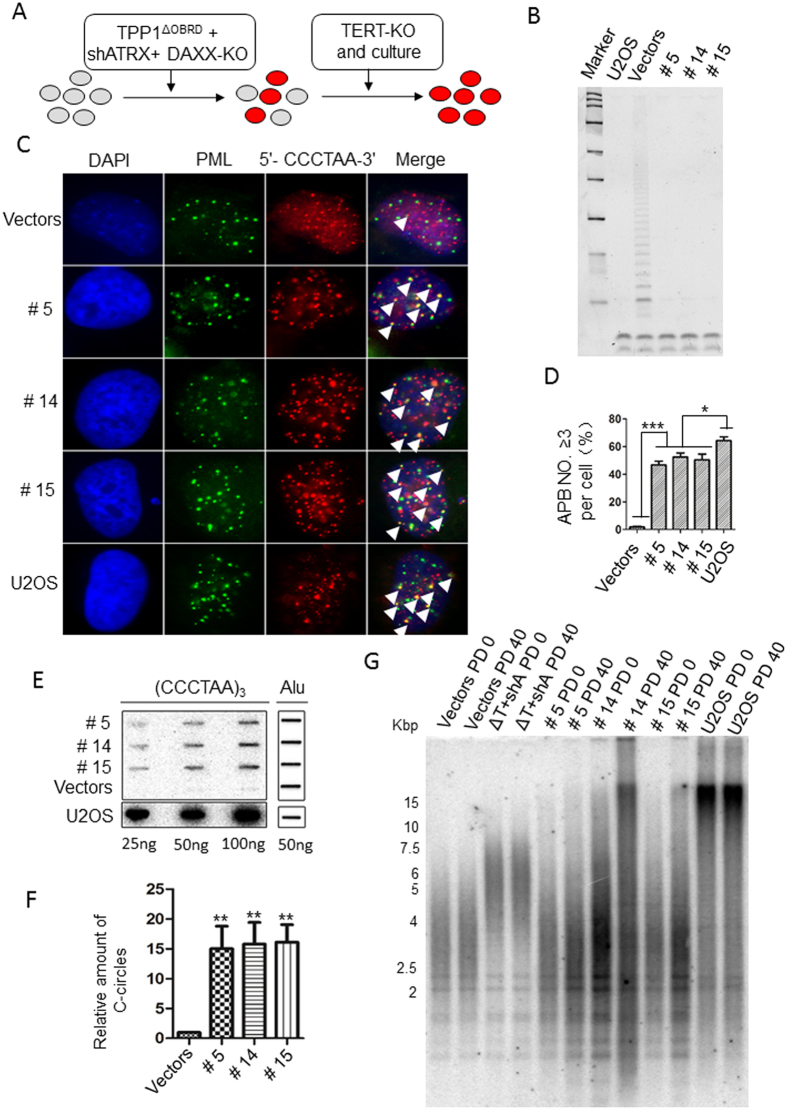
The detection of ALT activity in the TPP1^ΔOBRD^, ATRX knockdown, DAXX knock out and hTERT knock out cell lines. (**A**) Strategy for the construction of the ALT-like cell lines. The red circles indicate the potentially transformed cells. (**B**) TRAP assay to detect telomerase activity in the TERT-KO clones. We used the ΔT + shA + D-KO cell line to construct three TERT KO clones #5, #14 and #15. The bands at the bottom represent the internal control. U2OS cells were used as the positive control and HTC75 cells with vectors were used as the negative control. (**C**) IF-FISH staining for the #5, #14 and #15 cell lines. The PML bodies are labelled green. The telomeric DNA was labelled with (5′-CCCTAA-3′) _3_ probes (red). The white arrowheads indicate the APBs (yellow). (**D**) Statistical quantification of (**C**). The error bars indicated the standard error (n = 3). *P < 0.05; ***P < 0.001. (**E**) C-circle assay were performed to detect C-circles. U2OS cells were used as the positive control. (**F**) Statistical quantification of (**E**). The final relative amount of c-circles is the relative amount to vectors. The error bars indicate the standard error (n = 3). **P < 0.01. (**G**) Telomere length was detected using a TRF assay for different cell lines at PD0 and PD40. HTC75 cells with vectors were used as the negative control. U2OS cells were used as the positive control. #5, #14 and #15 represent different ΔT + shA + D-KO + TERT-KO cell lines. ΔT, TPP1^ΔOBRD^; shA, ATRX shRNA2; D-KO, DAXX knock out; TERT-KO, hTERT knock out.

To determine whether the telomere elongation phenotype observed in the ALT-like cells was related to the ALT mechanism, we used the CRISPR/Cas9 technique to generate hTERT knock out (TERT KO) cell lines based on the ΔT + shA + D-KO 1 cell line (ΔT + shA + D-KO + TERT-KO) (Fig. 4A). In addition, we picked up 7–10 clonal cell lines from different hTERT KO groups (Vectors + TERT-KO cells, ΔT + shA + TERT-KO cells and shA + D-KO + TERT-KO cells) for further studies after confirming no telomerase activity in them by using Q-TRAP assay and PCR-based sequencing (Fig. S4A–E). After two months in culture, only three ΔT + shA + D-KO + TERT-KO cell lines (termed #5, #14 and #15) survived (Figs S4E and S5). We next confirmed that these cell lines had no telomerase activity by telomeric repeat amplification protocol (TRAP) (Fig. 4B). IF-FISH experiments showed that approximately 40% of the cells had significantly increased APBs (Fig. 4C,D) and C-circles (Fig. 4E,F). We evaluated telomere length in PD0 and PD40 cells by using a TRF assay. In ΔT + shA cells, telomere length was longer than the Vectors control, but it did not continuously elongate after 40 PD culture and it was not as heterogeneity as U2OS cells (Fig. 4G). In PD0 of all threeΔT + shA + D-KO + TERT-KO cell lines, we could see that telomere length of these cells were longer than the Vectors control, especially the #14 cell line. In PD 40, the #14 cell line showed very heterogeneity telomere signals, which was very similar to the U2OS cells (Fig. 4G). In the other two cell lines #5 and #15, their telomere lengths were very similar to the Vectors control in PD0. But in PD40, we could see much more signals in the upper part comparing with PD0 (Vectors, #5 and #15) and the PD40 Vectors’ group. These signals indicated that telomeres had been elongated and became heterogeneously in #5 and #15 cells during passages, and there were much more extreme long telomeres appearing in late passage of cells (Fig. 4G). These results confirmed that ΔT + shA + D-KO + TERT-KO cells appeared the main hallmark of ALT.

## Discussion

Studies have suggested that anti-telomerase cancer therapy might force telomerase activity cancer cells switch to the ALT mechanism^10^,^14^,^47^. To better understand the mechanism that leads to the telomerase-ALT switch in telomerase-positive cancer cells, we constructed different cell lines with specific telomeric DNA damage, ATRX knockdown, DAXX deletion or TERT KO. We showed that overexpression of TPP1^ΔOBRD^, ATRX knockdown, DAXX deletion and TERT KO in telomerase-positive HTC75 cancer cells could promote ALT phenotypes by increasing APBs and C-circles, elongating telomeres and producing heterogeneous telomeres, which were very similar to ALT cells.

As a member of shelterin/telosome complex, TPP1 plays a vital role in telomere protection and maintenance^44^,^48^,^49^,^50^. DDR was detected in TPP1 mutant (TPP1^ΔOBRD^) HTC75 cells^49^. Our data indicated that only overexpression of TPP1^ΔOBRD^ to induce specific telomeric DNA damages did not change the formation of APBs and C-circles in telomerase activity cancer cells. In another study also showed the similar results. Overexpression of TRF1-FokI fusion protein in cells could specifically mediate telomeric double-strand break, it could increase the hallmarks of ALT recombination, drive homology search and non-sister telomeric chromatin synthesis in ALT cells, but not in telomere-positive cells^26^.

One of the reasons might due to telomerase-positive cancer cells have functional ATRX/DAXX complex. This chromatin remodelling complex and the histone variant H3.3 has been shown to be important for ALT repression. Loss of the ATRX protein and mutations in the ATRX gene are observed in most ALT cells^33^. Dysfunction in the ATRX-DAXX complex impairs the heterochromatic state of the telomeres, possibly because a reduction in H3.3 incorporation, which leads to telomere destabilization, creates a recombinogenic nucleoprotein structure for homologous recombination (HR) at the telomeres and thereby facilitates the ALT development. Furthermore, depletion of the histone chaperone, ASF1, alters the level of histone H3 at telomeric chromatin, which has been shown to induce ALT in both primary and cancer cells. Moreover, continuously knocking down of ASF1 led to increase in the level of γH2A.X^20^. This suggests that telomere chromatin dysfunction, caused by mutations of ATRX, DAXX or ASF1, might be sufficient to induce the telomeric DDR and trigger ALT-like cells.

In our study, using overexpression of TPP1^ΔOBRD^ and double knocking down ATRX and DAXX by shRNAs, we observed APBs and C-circles increasing during the switch from telomerase-positive cancer cells to ALT-like cells. The telomere length in cells that overexpression of TPP1^ΔOBRD^ and double knocking down ATRX and DAXX was elongated comparing with other controls. Interestingly, telomere length changed in the cell line with TPP1^ΔOBRD^ and ATRX knocking down. It has been reported that TPP1^ΔOBRD^ can negatively regulate telomerase recruitment to telomeres and deletion of the ATRX/DAXX complex might also disrupt telomerase assembly and recruitment^35^,^39^,^49^. However, telomerase activity played very important role in ALT repression so that inhibition of telomerase activity might promote ALT induction^14^. We had detected the telomerase activity in different ΔT + shA + D-KO cell lines by TRAP, which might repress the ALT mechanism to elongate telomeres^15^,^22^,^23^,^25^. Therefore, we induced hTERT knock out to completely eliminate telomerase activity and observed a more accurate ALT-like phenotype with significant heterogeneous telomere length. These results showed the changes including DNA damage mediated by overexpressing TPP1^ΔOBRD^, deletion of ATRX and DAXX, and TERT KO in telomerase-positive cancer cells indeed led to a telomerase-ALT switch. In summary, our results could increase the understanding of the mechanism of ALT induction and telomerase-ALT switch in telomerase-positive cancer cells.

## Materials and Methods

### Vectors and cell lines

HTC75, 293T and U2OS cells were cultured in DMEM with 10% fetal bovine serum. 293T was used to package virus and HTC75 was used to construct stable cell lines. TPP1^ΔOBRD^ (∆T), truncated 1–330 amino acids of human TPP1, was cloned into phage-based tet-on lentiviral vector which have a C-terminal HA-flag tag and G418 resistance. TPP1 mutant overexpressed with 100 ng/ml DOX treatment. The target sequences of ATRX shRNA1 (sh590, shATRX-1) and ATRX shRNA2 (sh592, shATRX-2) were previously reported^33^ and were cloned into pCl-mU6 retroviral vectors with puromycin resistance. The target sequences of shGFP and DAXX shRNA1/2 (shDAXX-1 and shDAXX-2) were previously reported and were cloned into pCl-H1 lentiviral vectors using blasticidin (BSD)^35^. The sequences of shRNAs are:

ATRX shRNA1: CGACAGAAACTAACCCTGTAA

ATRX shRNA2: CCGGTGGTGAACATAAGAAAT

shGFP:5′-CACAAGCTGGAGTACAACT-3′

DAXX shRNA1: 5′-AAGGAGTTGGATCTCTCAGAA-3′

DAXX shRNA2: 5′-GGTAAAGATGGAGACAAGA-3′

The knockout cell lines were established using a transient transfection system (PX330 vectors) with guide RNA targeting DAXX or TERT^51^.

The gRNA sequences are:

DAXX: GATGTTGCAGAACTCCGCCG AGG

hTERT: GGCAGTCAGCGTCGTCCCC GGG

### Western blotting and immunofluorescence *in situ* hybridization (IF-FISH)

Western blotting and IF-FISH were carried out as previously described^39^,^52^. For IF-FISH, the cells were fixed with 4% paraformaldehyde (Sigma-Aldrich, P6148), and then permeabilized with permeabilization buffer (0.5% triton X-100, 20 mM HEPES, 50 mM NaCl, 3 mM MgCl_2_, 300 mM sucrose)(Sigma-Aldrich,USA), blocked with 5% goat serum (Gibco, 16210–072) and incubated with primary and secondary antibodies. After incubation, the cells were fixed with 4% paraformaldehyde, then washed with. PBS and dehydrated with ethanol, followed by hybridization with a PNA-TelC-FITC probe (F1002,1:500,Panagene) and FITC-labeled (TTAGGG) peptide nucleic acid (PNA) probe (Panagene, Korea). Figs 1B and 2A used TTAGGG probe and other FISH analysis was performed by telomeric probe CCCTAA. More than 300 cells were counted in each cell line for APB scoring. The following antibodies and their working concentrations were used for Western blotting: anti-Flag (F7425, 1:8000, Sigma-Aldrich); anti-DAXX (sc-7152, 1:400, Santa Cruz Biotechnology); anti-ATRX (sc-15408, 1:400, Santa Cruz Biotechnology); anti-GAPDH (M20006M, 1:6000, Abmart). The following antibodies and their working concentrations were used for IF-FISH: anti-γH2AX (05–636, 1:500, EMD Millipore) and anti-PML (sc-966, 1:400, Santa Cruz Biotechnology).

### C-circles assay

The C-circles assay (CC assay) was carried out as previously described^43^. The cells used for C-circles assay are twenty population-doubling (PD20) after cell line constructed. A serial dilution of genomic DNA (100 ng, 50 ng and 25 ng) was used for hybridization with the probe of 5′-CCCTAA-3′ and 50 ng of genomic DNA were used for hybridization with Alu probe. The final relative amount of c-circles is the relative amount to shV/vectors. Unpaired t test was performed to calculate p-values.

### Telomeric Terminal Restriction Fragment (TRF) assay

The TRF assay was performed as previously described^50^. Normally, the cells used for C-circles assay are PD20 after cell lines constructed, and the cells for Fig. 4G are PD0 or PD40. The genomic DNA from different cells was quantified and the probe of 5′-CCCTAA-3′ was used for TRF assay.

### Telomeres repeat amplification protocol (TRAP) and Q-TRAP

TRAP assay was carried out as previously described^49^. Briefly, after the cells were harvested and counted, 10^5^ cells were washed with ice-cold PBS and the pellet was suspended with 100 μl of ice-cold lysis buffer (97.3 μl of DEPC-treated water, 1.7 μl of 1 mg/ml PMSF and 1 μl of 500 mM β-ME). The samples were incubated on ice for 30 min and then diluted to a suitable concentration for the assay. Telomerase activity was detected using a telomerase detection kit (EMD Millipore). The samples were run on an 8% polyacrylamide gel to confirm the PCR results. Q-TRAP assay was performed as previously described^35^. The probe of 5′-GGTTAG-3′ was used for Q-TRAP assay.

### Statistical analysis

All data are presented as the mean ± SD. The statistical significance of the difference between the mean values for the different genotypes was examined using unpaired two-tailed t-test. The data were considered significant when P < 0.05 (*), P < 0.01(**), P < 0.001(***). Otherwise, it would be considered not significant (N.S.).

## Additional Information

**How to cite this article**: Hu, Y. *et al*. Switch telomerase to ALT mechanism by inducing telomeric DNA damages and dysfunction of ATRX and DAXX. *Sci. Rep.* **6**, 32280; doi: 10.1038/srep32280 (2016).

## Supplementary Material

Supplementary Information

## References

[b1] d’Adda di Fagagna F. . A DNA damage checkpoint response in telomere-initiated senescence. Nature 426, 194–198 (2003). 14608368 10.1038/nature02118

[b2] Harley C. B., Futcher A. B. & Greider C. W. Telomeres shorten during ageing of human fibroblasts. Nature 345, 458–460 (1990). 2342578 10.1038/345458a0

[b3] Reddel R. R. Alternative lengthening of telomeres, telomerase, and cancer. Cancer Lett 194, 155–162 (2003). 12757973 10.1016/s0304-3835(02)00702-4

[b4] Blackburn E. H. Telomerases. Annu Rev Biochem 61, 113–129 (1992). 1497307 10.1146/annurev.bi.61.070192.000553

[b5] Bryan T. M., Englezou A., Dalla-Pozza L., Dunham M. A. & Reddel R. R. Evidence for an alternative mechanism for maintaining telomere length in human tumors and tumor-derived cell lines. Nat Med 3, 1271–1274 (1997). 9359704 10.1038/nm1197-1271

[b6] Morin G. B. The human telomere terminal transferase enzyme is a ribonucleoprotein that synthesizes TTAGGG repeats. Cell 59, 521–529 (1989). 2805070 10.1016/0092-8674(89)90035-4

[b7] Blackburn E. H. . Recognition and elongation of telomeres by telomerase. Genome 31, 553–560 (1989). 2698831 10.1139/g89-104

[b8] Ouellette M. M., Wright W. E. & Shay J. W. Targeting telomerase-expressing cancer cells. J Cell Mol Med 15, 1433–1442 (2011). 21332640 10.1111/j.1582-4934.2011.01279.x PMC3370414

[b9] Shay J. W., Reddel R. R. & Wright W. E. Cancer. Cancer and telomeres--an ALTernative to telomerase. Science 336, 1388–1390 (2012). 22700908 10.1126/science.1222394

[b10] Bechter O. E., Zou Y., Walker W., Wright W. E. & Shay J. W. Telomeric recombination in mismatch repair deficient human colon cancer cells after telomerase inhibition. Cancer Res 64, 3444–3451 (2004). 15150096 10.1158/0008-5472.CAN-04-0323

[b11] Sood A. K. . p53 null mutations are associated with a telomerase negative phenotype in ovarian carcinoma. Cancer Biol Ther 1, 511–517 (2002). 12496479 10.4161/cbt.1.5.167

[b12] Henson J. D. . A robust assay for alternative lengthening of telomeres in tumors shows the significance of alternative lengthening of telomeres in sarcomas and astrocytomas. Clin Cancer Res 11, 217–225 (2005). 15671549

[b13] Villa R. . Multiple mechanisms of telomere maintenance exist and differentially affect clinical outcome in diffuse malignant peritoneal mesothelioma. Clin Cancer Res 14, 4134–4140 (2008). 18593991 10.1158/1078-0432.CCR-08-0099

[b14] Hu J. . Antitelomerase therapy provokes ALT and mitochondrial adaptive mechanisms in cancer. Cell 148, 651–663 (2012). 22341440 10.1016/j.cell.2011.12.028 PMC3286017

[b15] Cerone M. A., Londono-Vallejo J. A. & Bacchetti S. Telomere maintenance by telomerase and by recombination can coexist in human cells. Hum Mol Genet 10, 1945–1952 (2001). 11555631 10.1093/hmg/10.18.1945

[b16] Heaphy C. M. . Prevalence of the alternative lengthening of telomeres telomere maintenance mechanism in human cancer subtypes. Am J Pathol 179, 1608–1615 (2011). 21888887 10.1016/j.ajpath.2011.06.018 PMC3181356

[b17] Flynn R. L. . Alternative lengthening of telomeres renders cancer cells hypersensitive to ATR inhibitors. Science 347, 273–277 (2015). 25593184 10.1126/science.1257216 PMC4358324

[b18] Yeager T. R. . Telomerase-negative immortalized human cells contain a novel type of promyelocytic leukemia (PML) body. Cancer Res 59, 4175–4179 (1999). 10485449

[b19] Cesare A. J. & Griffith J. D. Telomeric DNA in ALT cells is characterized by free telomeric circles and heterogeneous t-loops. Mol Cell Biol 24, 9948–9957 (2004). 15509797 10.1128/MCB.24.22.9948-9957.2004 PMC525488

[b20] O’Sullivan R. J. . Rapid induction of alternative lengthening of telomeres by depletion of the histone chaperone ASF1. Nat Struct Mol Biol 21, 167–174 (2014). 24413054 10.1038/nsmb.2754 PMC3946341

[b21] Napier C. E. . ATRX represses alternative lengthening of telomeres. Oncotarget 6, 16543–16558 (2015). 26001292 10.18632/oncotarget.3846 PMC4599288

[b22] Perrem K. . Repression of an alternative mechanism for lengthening of telomeres in somatic cell hybrids. Oncogene 18, 3383–3390 (1999). 10362359 10.1038/sj.onc.1202752

[b23] Perrem K., Colgin L. M., Neumann A. A., Yeager T. R. & Reddel R. R. Coexistence of alternative lengthening of telomeres and telomerase in hTERT-transfected GM847 cells. Mol Cell Biol 21, 3862–3875 (2001). 11359895 10.1128/MCB.21.12.3862-3875.2001 PMC87050

[b24] Katoh M., Kameyama M., Kugoh H., Shimizu M. & Oshimura M. A repressor function for telomerase activity in telomerase-negative immortal cells. Mol Carcinog 21, 17–25 (1998). 9473768 10.1002/(sici)1098-2744(199801)21:1<17::aid-mc4>3.0.co;2-m

[b25] Ford L. P. . Telomerase can inhibit the recombination-based pathway of telomere maintenance in human cells. The Journal of biological chemistry 276, 32198–32203 (2001). 11395519 10.1074/jbc.M104469200

[b26] Cho N. W., Dilley R. L., Lampson M. A. & Greenberg R. A. Interchromosomal homology searches drive directional ALT telomere movement and synapsis. Cell 159, 108–121 (2014). 25259924 10.1016/j.cell.2014.08.030 PMC4177039

[b27] Cesare A. J. . Spontaneous occurrence of telomeric DNA damage response in the absence of chromosome fusions. Nat Struct Mol Biol 16, 1244–1251 (2009). 19935685 10.1038/nsmb.1725

[b28] Chin L. . p53 deficiency rescues the adverse effects of telomere loss and cooperates with telomere dysfunction to accelerate carcinogenesis. Cell 97, 527–538 (1999). 10338216 10.1016/s0092-8674(00)80762-x

[b29] Chang S., Khoo C. M., Naylor M. L., Maser R. S. & DePinho R. A. Telomere-based crisis: functional differences between telomerase activation and ALT in tumor progression. Genes Dev 17, 88–100 (2003). 12514102 10.1101/gad.1029903 PMC195968

[b30] Heaphy C. M. . Altered telomeres in tumors with ATRX and DAXX mutations. Science 333, 425 (2011). 21719641 10.1126/science.1207313 PMC3174141

[b31] Goldberg A. D. . Distinct factors control histone variant H3.3 localization at specific genomic regions. Cell 140, 678–691 (2010). 20211137 10.1016/j.cell.2010.01.003 PMC2885838

[b32] Bower K. . Loss of wild-type ATRX expression in somatic cell hybrids segregates with activation of Alternative Lengthening of Telomeres. PLoS One 7 (2012). 10.1371/journal.pone.0050062 PMC3502299 23185534

[b33] Lovejoy C. A. . Loss of ATRX, genome instability, and an altered DNA damage response are hallmarks of the alternative lengthening of telomeres pathway. PLoS Genet 8 (2012). 10.1371/journal.pgen.1002772 PMC3400581 22829774

[b34] Clynes D. . Suppression of the alternative lengthening of telomere pathway by the chromatin remodelling factor ATRX. Nat Commun 6, 7538 (2015). 26143912 10.1038/ncomms8538 PMC4501375

[b35] Tang M. . Disease mutant analysis identifies a new function of DAXX in telomerase regulation and telomere maintenance. J Cell Sci 128, 331–341 (2015). 25416818 10.1242/jcs.159467 PMC4294776

[b36] Schwartzentruber J. . Driver mutations in histone H3.3 and chromatin remodelling genes in paediatric glioblastoma. Nature 482, 226–231 (2012). 22286061 10.1038/nature10833

[b37] Lewis P. W., Elsaesser S. J., Noh K. M., Stadler S. C. & Allis C. D. Daxx is an H3.3-specific histone chaperone and cooperates with ATRX in replication-independent chromatin assembly at telomeres. Proc Natl Acad Sci USA 107, 14075–14080 (2010). 20651253 10.1073/pnas.1008850107 PMC2922592

[b38] Zhong F. L. . TPP1 OB-fold domain controls telomere maintenance by recruiting telomerase to chromosome ends. Cell 150, 481–494 (2012). 22863003 10.1016/j.cell.2012.07.012 PMC3516183

[b39] Wan M., Qin J., Songyang Z. & Liu D. OB fold-containing protein 1 (OBFC1), a human homolog of yeast Stn1, associates with TPP1 and is implicated in telomere length regulation. J Biol Chem 284, 26725–26731 (2009). 19648609 10.1074/jbc.M109.021105 PMC2785360

[b40] Gossen M. & Bujard H. Tight control of gene expression in mammalian cells by tetracycline-responsive promoters. Proc Natl Acad Sci USA 89, 5547–5551 (1992). 1319065 10.1073/pnas.89.12.5547 PMC49329

[b41] Chung I., Osterwald S., Deeg K. I. & Rippe K. PML body meets telomere: the beginning of an ALTernate ending? Nucleus 3, 263–275 (2012). 22572954 10.4161/nucl.20326 PMC3414403

[b42] Osterwald S. . PML induces compaction, TRF2 depletion and DNA damage signaling at telomeres and promotes their alternative lengthening. J Cell Sci 128, 1887–1900 (2015). 25908860 10.1242/jcs.148296

[b43] Henson J. D. . DNA C-circles are specific and quantifiable markers of alternative-lengthening-of-telomeres activity. Nat Biotechnol 27, 1181–1185 (2009). 19935656 10.1038/nbt.1587

[b44] Chen L. Y., Liu D. & Songyang Z. Telomere maintenance through spatial control of telomeric proteins. Mol Cell Biol 27, 5898–5909 (2007). 17562870 10.1128/MCB.00603-07 PMC1952115

[b45] Wilkie A. O., Lamb J., Harris P. C., Finney R. D. & Higgs D. R. A truncated human chromosome 16 associated with alpha thalassaemia is stabilized by addition of telomeric repeat (TTAGGG)n. Nature 346, 868–871 (1990). 1975428 10.1038/346868a0

[b46] Wen J., Cong Y. S. & Bacchetti S. Reconstitution of wild-type or mutant telomerase activity in telomerase-negative immortal human cells. Hum Mol Genet 7, 1137–1141 (1998). 9618172 10.1093/hmg/7.7.1137

[b47] Queisser A., Heeg S., Thaler M., von Werder A. & Opitz O. G. Inhibition of telomerase induces alternative lengthening of telomeres during human esophageal carcinogenesis. Cancer Genet 206, 374–386 (2013). 24331919 10.1016/j.cancergen.2013.10.001

[b48] Kibe T., Osawa G. A., Keegan C. E. & de Lange T. Telomere protection by TPP1 is mediated by POT1a and POT1b. Mol Cell Biol 30, 1059–1066 (2010). 19995905 10.1128/MCB.01498-09 PMC2815557

[b49] Xin H. . TPP1 is a homologue of ciliate TEBP-beta and interacts with POT1 to recruit telomerase. Nature 445, 559–562 (2007). 17237767 10.1038/nature05469

[b50] O’Connor M. S., Safari A., Xin H., Liu D. & Songyang Z. A critical role for TPP1 and TIN2 interaction in high-order telomeric complex assembly. Proc Natl Acad Sci USA 103, 11874–11879 (2006). 16880378 10.1073/pnas.0605303103 PMC1567669

[b51] Cong L. . Multiplex genome engineering using CRISPR/Cas systems. Science 339, 819–823 (2013). 23287718 10.1126/science.1231143 PMC3795411

[b52] Liu D. . PTOP interacts with POT1 and regulates its localization to telomeres. Nat Cell Biol 6, 673–680 (2004). 15181449 10.1038/ncb1142

