# Supplementary material for: Switch telomerase to ALT mechanism by inducing telomeric DNA damages and dysfunction of ATRX and DAXX
Source: Sci Rep. 2016 Aug 31;6:32280. doi: 10.1038/srep32280 (PMC5006076; doi:10.1038/srep32280)
Supplement: Supplementary Information [file srep32280-s1.pdf]

**Supplementary Figures**

# Switch telomerase to ALT mechanism by inducing telomeric DNA damages and dysfunction of ATRX and DAXX

Yang Hu[1,2,#], Guang Shi[1,2,#,]*, Laichen Zhang[1], Feng Li[1], Yuanling Jiang[1], Shuai Jiang[1], Wenbin Ma[1], Yong Zhao[1], Zhou Songyang[1,2], Junjiu Huang[1,2,]*

# Supplementary Figure Legends

## Figure S1 Western blotting analysis in different cells.

(A) The expression level of Flag tagged TPP1$^{\Delta OBRD}$ (Flag-ΔT) was showed when the cells were treated with or without 100 ng/ml of DOX. GAPDH was used as the loading control.

(B) The knockdown efficiency of DAXX shRNA1 (shDAXX-1) and DAXX shRNA2 (shDAXX-2) were measured using western blotting. GAPDH was used as the loading control.

(C) The knockdown efficiency of ATRX and DAXX in the different cell lines was detected using western blotting. GAPDH was used as the loading control.

## Figure S2 The sequencing results of DAXX knock out clonal cell lines.

The sequencing results of DAXX knock out (KO) clonal cell lines. Red color represented base mutants or indels (insertion or deletion). KO2 represents one allele was deleted 417bp. WT, wild type.

## Figure S3 Examination of telomerase activity in the different cell lines.

(A) and (B) telomere repeat amplification protocol (TRAP) assay was performed in the different cell lines to detect telomerase activity. The bands at the bottom are internal control. U2OS was a negative control and HTC75 with vectors was a positive control. Flag-ΔT, Flag tagged TPP1$^{\Delta OBRD}$; shA, ATRX shRNA2; shV, Vector shRNA; shD1, DAXX shRNA-1; shD2, DAXX shRNA-2; D-KO, DAXX knock out.

## Figure S4 Construction of hTERT knock out (TERT-KO) cell lines.

(A) to (D), Q-TRAP was performed to confirm telomerase activity in the different clonal cell lines. Different TERT-KO clones including Vectors+TERT-KO, ΔT+shA+TERT-KO and ΔT+shA+D-KO+TERT-KO). RTA is short for relative telomerase activity. The numbers on the X-axis are stand for the different cell clones. (E) The table represented TERT-KO clones from different cell lines and the final numbers of survived clones after 2 months in culture. The number of TERT-KO clones which was confirmed by Q-TRAP. Survived clones did not have telomerase activity. *, ΔT+shA+D-KO+TERT-KO survived clonal cell lines were named as # 5, # 14, # 15.

ΔT, TPP1$^{\Delta OBRD}$; shA, ATRX shRNA2; D-KO, DAXX knock out; TERT-KO, TERT knock out.

## Figure S5 The sequencing results of DAXX or hTERT knock out clonal cell lines.

The sequencing results of TERT knock out (KO) clonal cell lines. Red lines represented base mutants (deletion). TPP1$^{\Delta OBRD}$, shATRX, DAXX-KO and TERT-KO survived clonal cell lines were named as # 5, # 14, # 15. WT, wild type.

# Supplement

## Figure S1

A

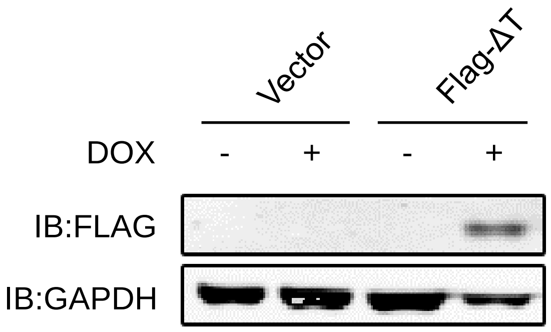


B

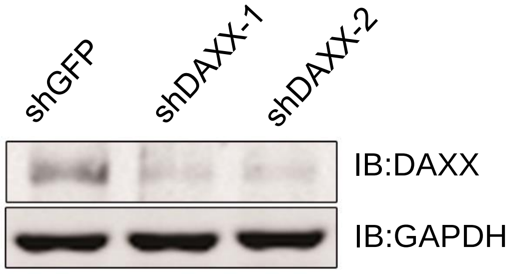


C

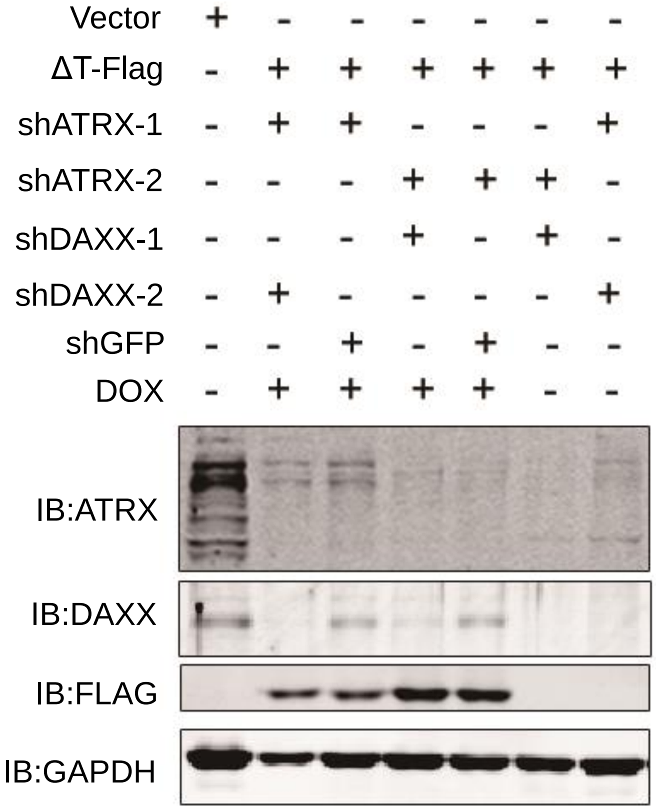

Figure S2

Sequencing results of DAXX-KO :

WT 5’- .. GATGTTGCAGAACTCCGCCG AGG ..-3’
5’- .. GATGTTGCAGAACTCCGCCG AGG ..-3’

KO1 GATGTTGCAGAACTCC-CCG AGG

GATGTTGCAGAACTCCG-CG AGG

KO2 GATGTTGCAGAACTCC-CCG AGG
—————————————————- (417bp deletion)

KO3 GATGTTGCAGAACTCCGGCCG AGG
GATGTTGCAGAACTCCGCGCCG AGG

Figure S3

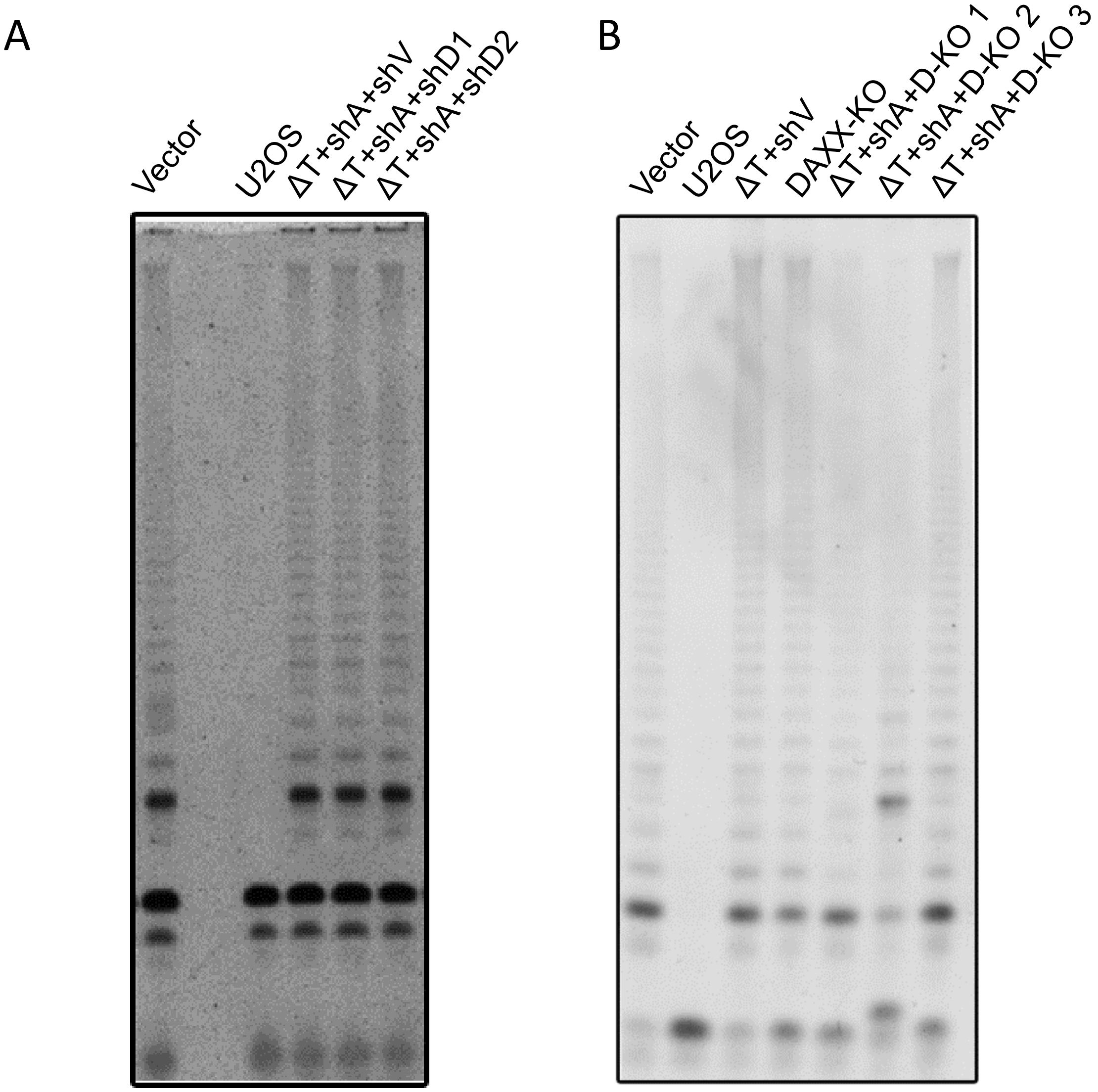
A
Vector
U2OS
ΔT+shA+shV
ΔT+shA+shD1
ΔT+shA+shD2
B
Vector
U2OS
ΔT+shV
DAXX-KO
ΔT+shA+D-KO 1
ΔT+shA+D-KO 2
ΔT+shA+D-KO 3

Figure S4

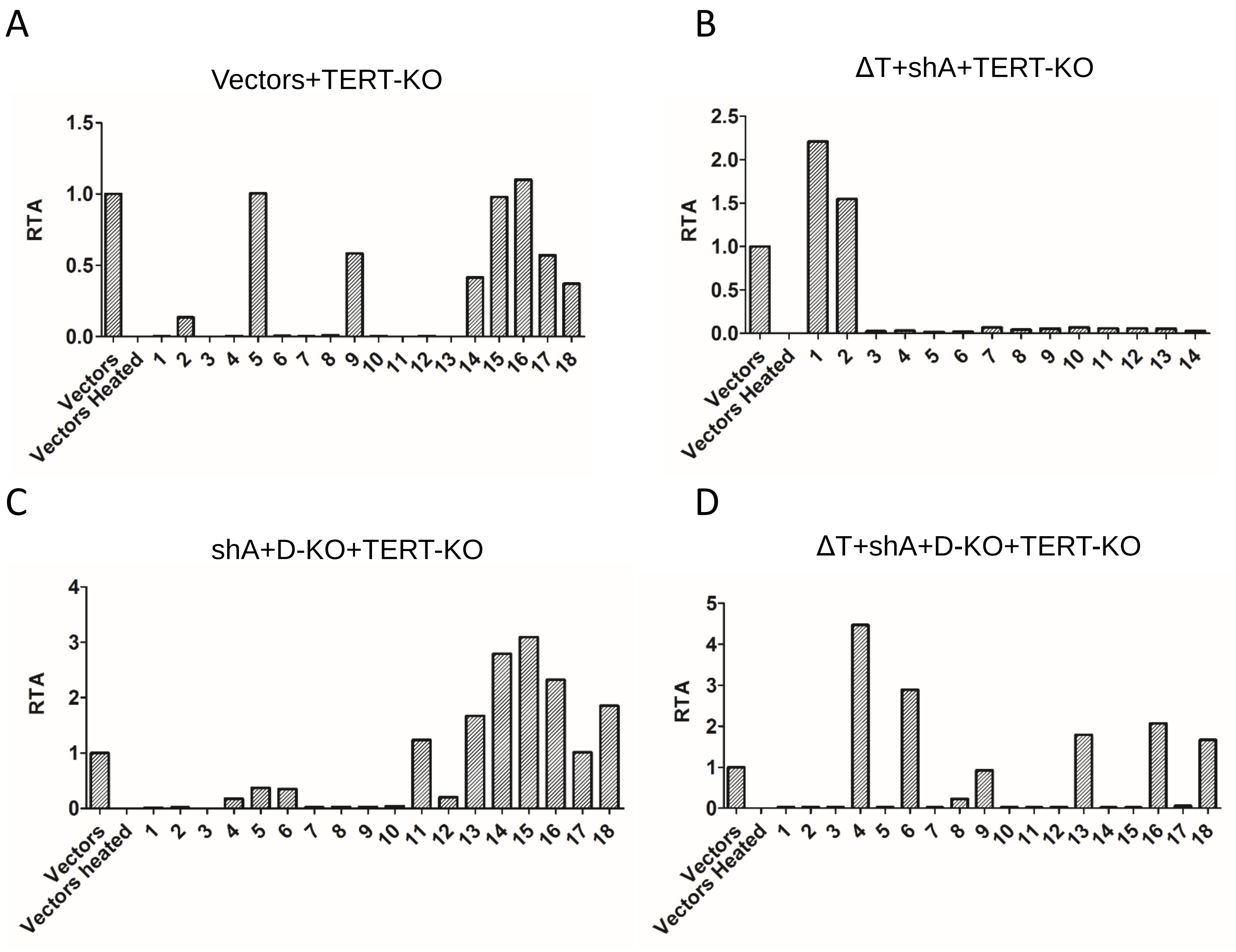


E

| Cell lines | TERT-KO clones | Survived clones （2 months culture） |
|---|---|---|
| Vectors+TERT-KO | 10 | 0 |
| ΔT+shA+TERT-KO | 10 | 0 |
| shA+D-KO+TERT-KO | 7 | 0 |
| ΔT+shA+D-KO+TERT-KO | 10 | 3* |

$TPP1^{\Delta OBRD}$, ΔT; shA, shATRX-2; D-KO, DAXX knock out

*, ΔT+shA+D-KO+TERT-KO survived clonal cell lines were named as # 5, # 14, # 15.

Figure S5

Sequencing results of hTERT-KO :

```
WT  5’- .. GGCAGTCAGCGTCGTCCCC GGG ..-3’
    5’- .. GGCAGTCAGCGTCGTCCCC GGG..-3’

#5         GGCAG--A-CG-----CCC GGG
           GGCAG-----------CCC GGG

#14        GGCAGTCAGCGTCGT-CCC GGG
           GGCAG--A-CG-----CCC GGG

#15        GGCAGTCAGCGTCGT-CCC GGGA
           GGC----------------   ---A
```
